# Frequency-based time-series gene expression recomposition using PRIISM

**DOI:** 10.1186/1752-0509-6-69

**Published:** 2012-06-15

**Authors:** Bruce A Rosa, Yuhua Jiao, Sookyung Oh, Beronda L Montgomery, Wensheng Qin, Jin Chen

**Affiliations:** 1 Biorefining Research Initiative and Department of Biology, Lakehead University, 955 Oliver Road, Thunder Bay, ON, P7B 5E1, Canada; 2 MSU-DOE Plant Research Laboratory, Plant Biology Laboratories, Michigan State University, 612 Wilson Road, Rm. 106, East Lansing, MI, 48824, USA; 3 Department of Biochemistry and Molecular Biology, Michigan State University, 603 Wilson Road, Room 212, East Lansing, MI, 48824, USA; 4 Department of Computer Sciences and Engineering, Michigan State University, 3115 Engineering Building, East Lansing, MI, 48824, USA

## Abstract

**Background:**

Circadian rhythm pathways influence the expression patterns of as much as 31% of the *Arabidopsis* genome through complicated interaction pathways, and have been found to be significantly disrupted by biotic and abiotic stress treatments, complicating treatment-response gene discovery methods due to clock pattern mismatches in the fold change-based statistics. The PRIISM (Pattern Recomposition for the Isolation of Independent Signals in Microarray data) algorithm outlined in this paper is designed to separate pattern changes induced by different forces, including treatment-response pathways and circadian clock rhythm disruptions.

**Results:**

Using the Fourier transform, high-resolution time-series microarray data is projected to the frequency domain. By identifying the clock frequency range from the core circadian clock genes, we separate the frequency spectrum to different sections containing treatment-frequency (representing up- or down-regulation by an adaptive treatment response), clock-frequency (representing the circadian clock-disruption response) and noise-frequency components. Then, we project the components’ spectra back to the expression domain to reconstruct isolated, independent gene expression patterns representing the effects of the different influences.

By applying PRIISM on a high-resolution time-series *Arabidopsis* microarray dataset under a cold treatment, we systematically evaluated our method using maximum fold change and principal component analyses. The results of this study showed that the ranked treatment-frequency fold change results produce fewer false positives than the original methodology, and the 26-hour timepoint in our dataset was the best statistic for distinguishing the most known cold-response genes. In addition, six novel cold-response genes were discovered. PRIISM also provides gene expression data which represents only circadian clock influences, and may be useful for circadian clock studies.

**Conclusion:**

PRIISM is a novel approach for overcoming the problem of circadian disruptions from stress treatments on plants. PRIISM can be integrated with any existing analysis approach on gene expression data to separate circadian-influenced changes in gene expression, and it can be extended to apply to any organism with regular oscillations in gene expression patterns across a large portion of the genome.

## Background

**Figure 1:**
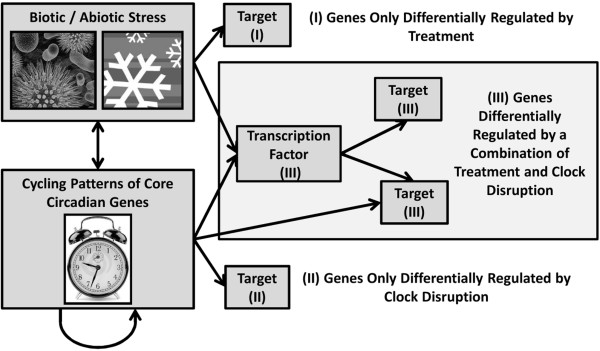
**Biotic and abiotic stresses both directly and indirectly influence target gene expression patterns**. Genes found to be differentially expressed may be influenced by (I) only direct treatment influences, (II) only indirect circadian-clock disruption influences, or (III) both direct treatment response and indirect clock influences.

Differential gene expression studies typically use the fold change statistic (the ratio of mRNA quantities between two samples) as input, and have been used to discover genes involved in adaptive stress responses which have not been previously characterized (i.e., “novel genes”) [1]. Specifically, to correct for changes in gene expression induced by non-treatment related influences, fold-change values for time-series data are usually calculated using treatment and control data at every timepoint [1]. One of the major factors causing gene oscillations under control conditions is the molecular circadian clock, which influences physiology and metabolism in preparation for predictable changes in light and temperature [2]. However, a wide range of biotic and abiotic stress treatments have been shown to disrupt rhythmic clock patterns through amplitude changes or phase shifts [3-8], resulting in significant fold changes for genes which are clock-influenced but are not involved in direct stress response. Figure1 demonstrates that genes can be differentially regulated due to direct stress responses (I), indirectly differentially regulated through disruption of clock pathways induced by the stress (II) or a combination of both (III). Additional complications in regulation patterns arise from the complexity of transcription factor pathways, in which targets may be regulated by clock components directly or through interactions with their transcription factors (Figure1). For this reason, novel treatment-response gene discovery methods are complicated by the disruption of synchronization of the circadian rhythm pathways, but this complexity is not reflected in existing methods including fold change studies, clustering analysis approaches, and more complex time-serial-based algorithms [1,2,5,6,9-17].

**Figure 2:**
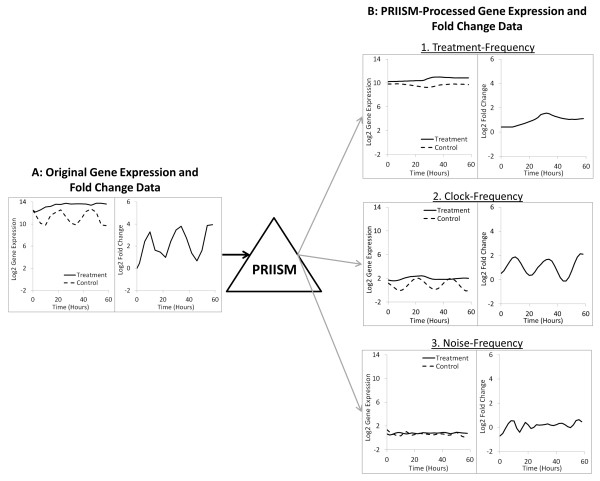
**PRIISM separates gene expression data into three independent gene expression datasets.** PRIISM separates (**A**) the original gene expression patterns under control and treatment conditions (used to calculate the fold change pattern) into **(B**) treatment-frequency, clock-frequency and noise-frequency gene expression patterns. The cold-induced gene *COR15A* (*AT2G42540*) is shown as an example.

In this paper, we present the PRIISM (Pattern Recomposition for the Isolation of Independent Signals in Microarray data) algorithm to perform novel stress-response gene discovery analyses which correct for differential gene expression patterns induced by the circadian clock. As described previously [6], although core circadian clock gene patterns undergo significant changes in phase and amplitude as a result of stress, they maintain oscillating frequencies which remain similar to each other, and still remain close to the circadian pattern of one cycle per day. It has also been shown that stress results in significantly increased average expression levels for stress-response genes [6], which are reflected in the low-frequency signals (where one oscillation cycle occurs over the course of several days) for these genes. We assume that although circadian clock influences and adaptive stress-response influences can interact with each other (Figure1), they still cycle at very different rates from each other (and therefore maintain separate dominant frequency ranges) under stress conditions. Based on these observations, we have developed PRIISM to project gene expression data to the frequency domain using the Fourier Transform, isolate independent signals, and then project them back to the expression domain to reconstruct independent gene expression patterns representing the effects of different genetic influences. PRIISM is capable of separating one gene expression pattern into three distinct gene expression patterns: (1) The treatment-frequency gene expression pattern, which has much of the complicating circadian influences removed, and consequently can be used to more accurately identify differentially regulated genes which are involved in direct treatment response, (2) the clock-frequency gene expression pattern, representing rhythmic patterns with a period of approximately one cycle per day, and (3) the noise-frequency gene expression pattern (Figure2). By applying PRIISM on a cold-treatment dataset, we demonstrate that it can identify known treatment-response genes with a much lower false-positive rate than the existing methods, and can also identify important regulatory timepoints which are not obvious in the unprocessed data. In addition to improving performance when conducting novel treatment-response gene discovery, PRIISM also provides gene expression data which represent only circadian clock influences, and may be useful for circadian clock analysis studies.

Biological approaches such as the use of constant light and clock component genetic knockout mutants are applied in order to attempt to remove the influences of the circadian clock on target gene expression. However, constant light is an unnatural condition which reduces the applicability of the results, because natural biotic and abiotic genetic stress-response patterns depend on the time-of-day (the point in the light/dark cycle) at which the treatment is applied [6,18,19]. Likewise, the use of genetic knockout mutants of circadian clock genes can reduce disruptions due to circadian input; However, since stress response genes may be regulated by clock components, the results of such a study are also difficult to interpret [7,19,20].

Most existing computational approaches for studying differential gene expression in microarray datasets involve clustering algorithms designed to group genes with similar expression profiles, with the goal of identifying potential annotations for unknown genes [10-17]. However, the gene distance measures used by all of these clustering methods are unable to distinguish adaptive-response gene expression patterns from circadian clock disruption gene expression patterns, and so may cluster genes with similar clock influences but very different treatment-response influences. Bar-Joseph et al’s (2003) continuous representation model for finding differentially expressed genes in time series micro array datasets (which has been used to find more cell-cycle response genes in yeast than conventional clustering methods) is also unable to filter clock influences from treatment response influences on gene expression patterns [21].

Several studies have shown that between 6% and 31% of the *Arabidopsis* genome is influenced by circadian clock genetic components [5,22,23]; while another study suggests that there are significant baseline circadian oscillations for nearly 100% of the genome [24]. A number of approaches have been developed for analyzing the circadian rhythms of genes in time-series datasets [5,25-28]. Fourier analysis (which can be used to identify dominant frequencies in time-series data) has been applied to successfully identify periodic genes by treating time-series microarray datasets as time-domain signals [28-32]. However, these Fourier analysis methods have not been widely used in differential gene expression study methods, because 1) in existing Fourier analysis applications [28-32], a fixed frequency range was used as *a priori* knowledge to discover genes with similar oscillations, but novel genes may have totally different frequency patterns under different treatment conditions and; 2) to accurately capture oscillating rhythms, high resolution time course gene expression data is essential according to *Nyquist sampling theorem*[25,33], but such data have not been available until recently.

As the price of running microarrays and RNA-seq chips continues to fall, high-resolution time-series gene expression datasets that contain enough information to identify and characterize circadian-frequency rhythms for every gene are becoming available [34-36]. Recently, Espinoza *et al.* (2010) produced one such microarray dataset, which measured 16 timepoints covering a 58-hour time period with a cold treatment in *Arabidopsis*[7]. Cold-stress genetic responses in *Arabidopsis* are particularly well-characterized, and have been shown to significantly dampen and phase-shift the oscillations of the core clock genes *CCA1* and *LHY*, which have regulatory influences over some cold-responsive transcription factors, including *CBF1CBF2* and *CBF3*[20]. Disruption of the expression patterns of other circadian output marker genes due to cold treatment has also been reported, including constant over expression of *CAB2* and *CCR2,* and constant underexpression of *CAT3*[6,9]. For these reasons, this is an ideal dataset to test whether the PRIISM algorithm is able to separate the strong circadian-clock influences on cold-response genes from treatment-response influences.

## Methods

A wide range of biotic and abiotic stress treatments have been shown to significantly disrupt the cyclic patterns of core circadian clock genes and their downstream target genes [3-8]. When a stress treatment is constantly applied, adaptive stress-response genes are expected to be differentially regulated, while influences from the circadian clock will cause oscillations in target gene expression patterns. In PRIISM, by projecting the gene expression data to the frequency domain using the Fourier transform [37], the resulting amplitude spectra peak at different frequencies, caused by these different influences. The Fourier transform is a mainstream signal processing technique that simplifies period gram analysis by identifying the dominant frequencies in the amplitude spectrum. By distinguishing the clock frequency range from the core circadian clock genes in the frequency domain, we can separate the spectrum to different sections containing treatment-related, clock-related and noise-related influences. Then, we project the amplitude spectra back to the expression domain to reconstruct isolated, independent gene expression patterns representing the effects of different frequency components. This method can be applied to any dataset which has sufficiently high resolution and length to measure frequencies of at least one cycle per day, and which uses a treatment which is applied at a frequency significantly different than the clock frequency.

**Figure 3:**
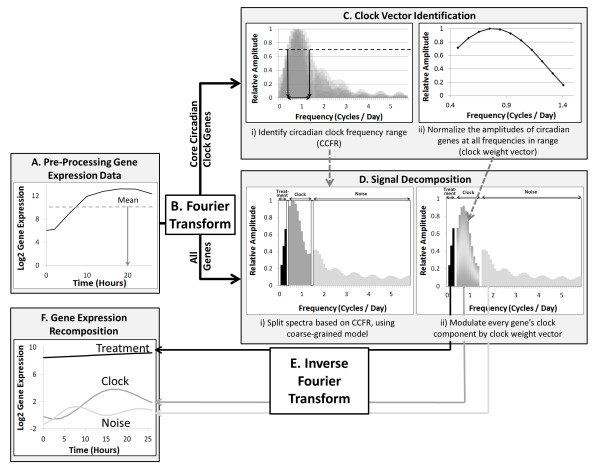
**Workflow of the PRIISM algorithm.** The 0 to 26 hour time-frame in the cold for *AtgolS3* (*AT1G09350*) is used as an example.

PRIISM has four steps (Figure3). In the first step, gene expression data are pre-processed to fit the requirements of the Fourier transform, after which the Fourier transform is performed to produce an amplitude spectrum for every gene (Figure3A, 3B). In the second step, a clock vector that defines the frequency range and the amplitudes of the core circadian clock genes is identified based on the spectra of core circadian clock genes (Figure3C). In the third step, the clock vector is used to decompose every gene’s spectrum into three components (treatment, clock and noise; Figure3D). In the final step, the inverse Fourier transform is applied to project each spectrum component back to the expression domain, resulting in three independent expression patterns (Figure3E, 3F).

### Pre-processing and Fourier analysis

Time series gene expression data are often unevenly sampled, and the disruption of clock patterns caused by the treatment varies over time. To be able to apply the Fourier transform (which requires steady and evenly sampled input), pre-processing is required. First, the whole time course is divided into overlapped frames. The size of these frames can be changed depending on the experiment; If they are too long, then it may be difficult to capture changes over time, and if they are too short, then it is more difficult to capture the treatment-frequency patterns (particularly for low-resolution data). For this experiment, the first time frame is 26 hours long due to the two-hour light period at the start of the time period, and all the other time frames are 24 hours long, starting and ending at each light/dark transition ( Additional file 1: Figure S1A). Second, within each time frame, the gene expression data is interpolated (making the time points evenly sampled), and the mean of the gene expression data for each gene is shifted to zero (refer to explanation of Eq. 2). The Fourier transform is then applied on each overlapping time frame individually, and the final expression values for each timepoint are calculated using a weighted average for each time frame, where higher weights are used for expression values near the center of each time frame ( Additional file 1: Figure S1B).

Fourier analysis is a signal processing technique [37] for the study of two processes: The Fourier transform (the process of decomposing a signal into a sum of components with different frequencies) and the inverse Fourier transform (the operation of reconstructing the signal from these components). Specifically, the discrete Fourier transform (DFT) and its inverse have been used to transform gene expression signals and to reconstruct the discrete signal, respectively [38]. The Fourier coefficient of DFT (*G*_*n*_) measures the contribution of the corresponding frequency component to the original signal and is given in Eq. 1 [37]:

(1) $$\begin{array}{cc} G_{n}=\sum_{k=0}^{K-1}g(kT)e^{-i2\pi \frac{n}{NT}k} & n=0,\ldots ,N-1 \end{array}$$

where, *g(kT)* is the sampled signal of *K* samples with the sampling interval *T*; *i* is the imaginary unit. The frequency of the corresponding component *n* is denoted as *f*_*n*_ (i.e.*,*$\frac{n}{NT}$), where *N* is the number of frequency components. The DFT maps a time course signal into the frequency domain by producing a spectrum. An amplitude spectrum (plotted as the amplitude versus frequency) is a common frequency domain representation of the original signal. Fast Fourier transform (FFT) is an efficient algorithm to compute the DFT and its inverse [37]. Because of its popularity, it has been built into most modern analysis tools including MATLAB and R [39,40].

The Fourier coefficient of the zero-frequency component (*G*_*0*_), derived from Eq. (1) where *f*_*n*_ = 0, is shown in Eq. 2 as given in [37]:

(2) $$G_{0}=\sum_{k=0}^{K-1}g(kT)e^{-i2\pi k*0}=\sum_{k=0}^{K-1}g(kT)$$

Note that there is a dominant peak at zero frequency in the spectrum of the expression value, which may bias the identification of the true dominant peak to frequency zero. To avoid such bias, we shift the mean of the time course gene expression values for each gene to zero (and consequently *G*_*0*_ *= 0*), leading to the removal of the peak at zero frequency. For example, the mean expression value for the gene shown in Figure3A is reduced from 10.6 to 0, and will be added back proportionally to the reconstructed gene expression values during signal recomposition.

### Identification of the circadian clock frequency range

The *Arabidopsis* circadian clock is composed of multiple feedback loops. Three genes, *Circadian ClockAssociated 1* (*CCA1*), *Late ElongatedHypocotyl* (*LHY*) and *Timing of CAB Expression 1* (*TOC1*) compose the first and most important feedback loop controlling the circadian clock, while *Pseudo Response Regulators 7* and *9* (*PRR7* and *PRR9*) form a secondary feedback loop with *CCA1* and *LHY*, and a third feedback loop involving *TOC1* is regulated by unknown components [41,42]. It has been found that through these feedback loops, eight core circadian clock genes (*CCA1LHYPRR7PRR9ELF4GILUX* and *TOC1*) and their downstream gene targets regulate a wide range of downstream pathways, including germination, leaf development, organelle morphology, photosynthesis, and cell wall development [2,18,43-46].

The Fourier transform is performed on these eight core circadian genes (Figure3Ci). The frequency components with relative amplitudes greater than 0.7 (corresponding to half of the maximum value in the spectra) are chosen as dominant frequencies [47]. We define the union of these eight sets of dominant frequencies as Circadian Clock Frequency Range (CCFR), noted as *f*_*c_min*_*f*_*c_max*_, where *f*_*c_min*_ is the lowest frequency, and *f*_*c_max*_ is the highest frequency (Figure3Ci). Note that in this example, the dominant clock frequency is significantly lower than one cycle per day, due to the stress-induced disruption of clock patterns. The weight of each frequency component in the CCFR is derived as:

(3) $$\begin{array}{cc} w_{n}=\frac{\sum_{m=1}^{8}{\left|G_{\mathrm{mn}}\right|}^{2}-\min(G)}{\max(G)-\min(G)} & n\in [c\_min,c\_max] \end{array}$$

where |*G*_*mn*_ | is the magnitude of the Fourier coefficient of the *n*_*th*_ frequency component for the *m*_*th*_ core circadian gene, $G=\left\{\sum_{m=1}^{8}{\left|G_{mc\_min}\right|}^{2},\sum_{m=1}^{8}{\left|G_{m(c\_min+1)}\right|}^{2},\ldots ,\sum_{m=1}^{8}{\left|G_{mc\_\max}\right|}^{2}\right\}$ is the set of the summed power of eight core clock genes present at each frequency component within the Circadian Clock Frequency Range (CCFR), and *w*_*n*_ is the weight for the frequency component at frequency *f*_*n*_. The vector {*w*_*c_min*_*w*_*c_min*+1_,…, *w*_*c_max*_} defines the gain-frequency response of a tapering bandpass filter within the CCFR.

### Signal decomposition and recomposition

We apply Fourier analysis on each gene, producing the relative amplitude spectrum from which we identify three distinct sections: Treatment-frequency, clock-frequency and noise-frequency components (Figure3Di). For the treatment-frequency decomposition, given a relatively narrow frequency band, we used a low pass filter with a steep cut-off frequency to gain the optimal balance between removing ringing artifact and approximating desired frequency response [48]. This issue is addressed in detail in the “Justification for choosing a steep cut-off frequency for the low-pass filter” section of the ( Additional file 1: Figure S3Figure S4 and Figure S5) [49].

The clock component is derived by bandpass filtering. Fourier coefficients of the clock components of each gene are modulated by the weight of the corresponding frequency components, as given by Eq. 4:

(4) $$\begin{array}{cc} {\hat{G}}_{c}=w_{c}G_{c} & c\in \left[c\_min,c\_max\right] \end{array}$$

The tapering filtering results in clock-frequency expression patterns that are noise reduced and with less artifacts caused by a discontinuity in the filter function. The reconstructed high frequency expression pattern is considered to be noise, and it is not studied in this paper. Therefore, we simply applied an ideal high pass filter. The reweighted spectra used for the signal reconstruction of the three frequency components sections are shown in Figure3Dii.

The inverse discrete Fourier transform (IDFT) is calculated according to Eq. 5 [37]:

(5) $$\begin{array}{cc} g(kT)=\frac{1}{N}\sum_{n=0}^{N-1}G_{n}e^{i2\pi \frac{n}{NT}k} & k=0,\ldots ,K-1 \end{array}$$

The inverse Fourier transform is performed on the full spectrum, including the filtered spectra for each gene. Similar to using the clock vector as a tapering band-pass filter to remove noise, we added a coarse graining process to make sure there is no overlapping between any of the two frequency bands, which may increase the robustness of component selection. The mean of the original gene expression values (which was removed in the pre-processing step), is added back proportionally to each gene expression curve based on the amplitude distribution of each component in the spectra before shifting the mean (Figure3F), according to Eq. 6:

(6) $$\begin{array}{cc} g_{L}^{'}(kT)=g_{L}(kT)+\frac{\sum_{k=0}^{K-1}g(kT)\sum {\left|G_{L}^{0}\right|}^{2}}{K\sum {\left|G_{n}^{0}\right|}^{2}} & \;k=0,\ldots ,K-1 \end{array}$$

where $g_{L}^{'}(kT)$ is the treatment expression level at timepoint *kT* for a given gene, $g_{L}^{}(kT)$ is a result of inverse discrete Fourier transform (Eq. 5) on treatment frequency at timepoint *kT*, *G*_*n*_^*0*^ is the Fourier coefficient of original gene expression values and *G*_*L*_^*0*^ is the Fourier coefficient of original gene expression values in the treatment-frequency band [*0,f*_*c_min*_*-1*]. Similarly, we compute $g_{C}^{'}(kT)$ and $g_{N}^{'}(kT)$ i.e., the clock expression level and noise level.

Note that because the entire warm and cold gene expression datasets are mean-shifted based on their relative amplitudes in each component, the reconstructed time-zero fold change values may not necessarily be equal to zero (Figure2B).

## Results and discussion

This study analyzes an *Arabidopsis* Affymetrix ATH1 microarray dataset (containing 22,810 probes) generated by Espinoza *et al.* (2010), which consists of 16 timepoints collected over the course of 58 hours in both warm (20 °C) and cold (4 °C) conditions under a 16-hour light/8-hour dark cycle starting at ZT14 (14 hours after dawn) [7]. This dataset was chosen for the analysis because it has separate control and treatment arrays, it has sufficiently high resolution (sampled at 2 hours and every 4 hours after that), and cold is a well-studied treatment in *Arabidopsis*[6,7,9,20,50,51].

Gene expression data was RMA normalized using the “affylmgui” program available as part of the *Bioconductor* software package and annotated using annotation data available from TAIR (version 10, available ftp://ftp.arabidopsis.org/Genes/TAIR10_genome_release/). The gene expression data were interpolated to every 2 hours using B-spline regression, and were segmented into four overlapping gene expression time frames (from both the warm and cold treatments), which were combined using a weighted average ( Additional file 1: Figure S1) [52,53]. PRIISM was applied on this “original” dataset, resulting in three independent and isolated gene expression datasets (treatment-frequency, clock-frequency and noise-frequency).

### Treatment-response gene discovery

**Figure 4:**
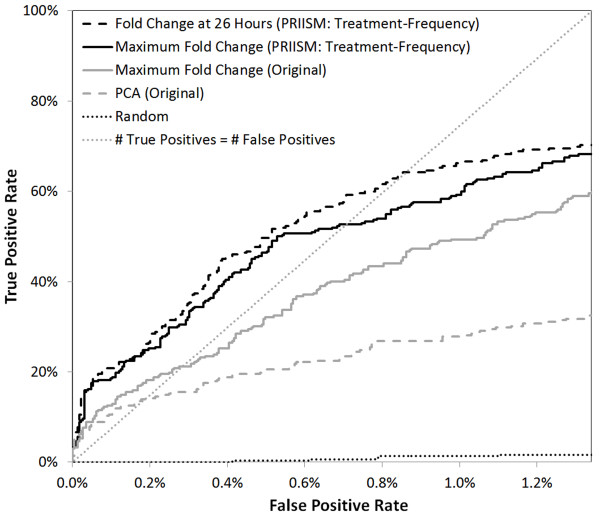
**ROC curves for COS-upregulated genes**. ROC Curves for the 26-hour treatment-frequency fold change (dashed black line), the treatment-frequency maximum fold change (solid black line), the original maximum fold change (solid grey line), and original PCA plot distance data (dashed grey line) are shown. The point at which the number of false positives is equal to the number of true positives (dotted grey line) and random gene selection (dotted black line) are also shown.

In order to show the advantage of PRIISM, we identified known cold-response genes using maximum fold changes and principal component analysis in the treatment-frequency dataset compared to the original dataset. Fold change values were calculated by subtracting the logged gene expression value in the warm from the logged gene expression value in the cold at every timepoint. Lists of *Arabidopsis* genes upregulated by cold treatment when grown on agar plates or grown in soil were collected from a previous study by Vogel et al [54]. The 302 cold-upregulated genes found in the intersection of these lists were used to define the set of “cold standard” (COS) upregulated genes. Receiver-Operator-Characteristic (ROC) curves (which have been shown to be an effective method for evaluating gene expression data [55]) were generated for these COS-upregulated genes [54] by distinguishing each ranked gene as either a true positive or a false positive (Figure4). A larger area under an ROC curve indicates that more COS-upregulated genes are identified. The line at which the number of true positives is equal to the number of false positives is indicated in Figure4, and only the data above this line are considered biologically relevant. By ranking genes by their maximum fold change values in the treatment-frequency dataset, 52.6% (159/302) of known COS upregulated genes can be identified, compared to only 21.2% (64/302) in the original dataset (Table1) [54]. This difference may be explained by the disruptions contributed by the clock-frequency influences and the noise-frequency influences, which are present in the original dataset. This shows that more COS-upregulated genes can be identified by ranking by the maximum fold change in the treatment-frequency dataset compared to the original dataset.

**Table 1:** Summary of ROC analysis for genes upregulated by cold treatment

Statistic	Original Data		Treatment-Frequency Data		
	Maximum Fold Change	PCA Distance	Maximum Fold Change	PCA Distance	Fold Change at 26 Hours
Recall when true positives = false positives	21.2%	13.9%	52.6%	46.0%	64.2%
Number of true positives identified when true positives = false positives (Out of 302 true positives)	64	42	159	139	194

Principal component analysis (PCA) is a linear component composition method that has been applied to summarize different gene expression influences under different conditions, and consequently has been used for differential gene expression studies in microarray datasets [56]. PCA was performed on the original dataset ( Additional file 1: Figure S2A), and the Euclidean distance from the bottom-left of the PCA plot of the first and second component was used to rank the genes, allowing for the construction of an ROC curve based on this data (Figure4). These data show that only 13.9% (42/302) of the cold upregulated genes can be identified in the original PCA plot. The first PCA components of the treatment-frequency data and the clock-frequency data were also plotted ( Additional file 1: Figure S2B) and ranking based on Euclidean distance from the bottom-right was able to identify 46.0% (139/302) of the COS-upregulated genes.

These results showed that, in both maximum fold change and PCA analyses, the ranked treatment-frequency fold change analyses produce fewer false positives than the original methodology by distinguishing more COS-upregulated genes (Table1).

### The identification of important gene regulation timepoints using PRIISM

In the previous section, it was shown that gene discovery in the treatment-frequency data produced by PRIISM constantly outperforms the same analyses on the original data. Although these approaches are useful for poorly studied treatment responses, a knowledge-based approach may be used to identify more treatment-response genes with a lower false positive rate.

Cold treatments have been shown to induce the expression of the transcription factors *C-repeat/DRE Binding Factor* genes *CBF1, CBF2* and *CBF3*[57]*,* which are induced in parallel with the cold transcription factors *RAV1* and *ZAT12*[50]. Some of the important targets of *CBF* transcription factors include *Cold-Responsive* (*COR*) genes *COR15A, COR15BCOR47,* and *COR78*[20,50,58,59]. All of the cold transcription factors and targets included in these lists have also been shown to be gated by the circadian clock, making them ideal for evaluating PRIISM’s ability to remove clock-frequency influences [6,20,23].

**Figure 5:**
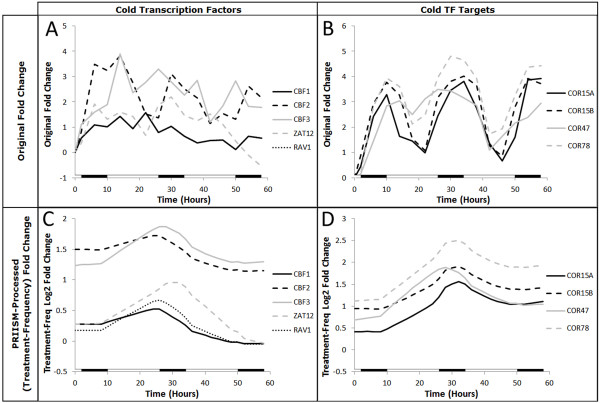
**Fold change patterns of cold transcription factors and target genes before and after PRIISM processing**. The original fold change patterns for important cold transcription factors (**A**) and some of their important target (COR) genes (**B**) are shown, along with their the treatment-frequency fold change patterns for the same genes (**C & D**). Thin, dashed vertical lines are used to indicate the 26 hour position on each graph.

In the treatment-frequency data, a peak in the fold change patterns can be observed in the well-studied cold response transcription factors and cold regulated (COR) response genes at the start of the first night (at approximately 26 hours) (Figure5C, 5D). The peaks of the transcription factors can be seen to occur before the peaks of their target genes, as is expected for a TF-target relationship. By contrast, these peaks are not apparent in the original fold change data (Figure5A, 5B). For this reason, an ROC curve was computed using the fold change value at 26 hours in the treatment-frequency fold change data (Figure4, Table1). Table1 shows that 194/302 (64.2%) of the true-positive COS-upregulated genes can be identified with a 50% false positive rate in the treatment-frequency 26-hour fold change data, compared to only 64/302 (21.2%) for the maximum fold change in original data and 42/302 (13.9%) for the PCA plot of the original data .

This data shows that the fold change value at 26 hours in the treatment-frequency data is the best predictor of whether a gene is involved in adaptive cold response. The top 25 ranked genes based on fold changes at 26 hours in the treatment-frequency dataset are shown in Table2. Included in this table is the “Cold Upregulation Category” for each gene, which indicates whether a gene was upregulated in the cold when plants were grown in soil (“Soil”), on agar plates (“Plate”), on both growth mediums (“COS”), or on neither (“Novel”) in Vogel et al’s study [54]. In this table, 22/25 of the genes belonged to the COS group, 2 belonged to the “Plate” group, and 1 belonged to the “Soil” group, suggesting that the PRIISM method has successfully identified known cold-regulation genes [54]. Table3 shows the top 25 ranked genes which were not part of the COS-upregulated gene list in Vogel et al (2005) [54]. 10/25 of the genes in this list belonged to the “Soil” group, 9 belonged to the “Plate” group, and 6 were novel genes not identified in Vogel et al’s study [54]. All of the novel genes (and all but one of the 25 genes in this list) have been previously identified as being involved in cold response in other studies, suggesting that PRIISM has identified a list of very important cold-response genes (See “Comments” column in Table 8).

**Table 2:** Genes ranked based on their treatment-frequency fold change values at 26 hours

**Rank**	**Annotation**	**P-Value (Treatment-Frequency Fold Change, 26 Hours)**	Cold Upregulation Category*[54]
1	*AT1G09350*: *Arabidopsis thaliana* Galactinol Synthase 3 (*AtGolS3*)	3.97E-31	COS
2	*AT4G14690*: Early Light-Inducible Protein 2 (*ELIP2*)	3.18E-28	COS
3	*AT4G12470*: Azelaic Acid Induced 1 (*AZI1*)	1.23E-25	COS
4	*AT3G50970*: Low Temperature-Induced 30 (*LTI30*)	3.62E-22	COS
5	*AT1G16850*: Unknown protein	7.38E-22	COS
6	*AT3G22840*: Early Light-Inducible Protein 1 (*ELIP1*)	8.66E-21	COS
7	*AT1G51090*: Heavy-metal-associated domain-containing	1.41E-16	COS
8	*AT3G55580*: Regulator of chromosome condensation (*RCC1*) family protein	3.62E-16	COS
9	*AT5G25110*: *CIPK25* (CBL-Interacting Protein Kinase 25)	1.13E-13	COS
10	*AT5G52310*: *COR78* (Cold Regulated 78)	3.25E-13	COS
11	*AT1G02820*: late embryogenesis abundant 3 family protein/LEA3 family protein	3.29E-13	Soil
12	*AT4G30830*: similar to unknown protein (*AT2G24140.1*)	5.57E-13	COS
13	*AT2G23910*: Cinnamoyl-CoA reductase-related	9.97E-13	Plate
14	*AT1G48100*: Glycoside hydrolase family 28 protein/polygalacturonase (pectinase) family protein	1.93E-12	COS
15	*AT5G17030*: UDP-Glucosyl Transferase 78D3 (*UGT78D3*)	3.07E-12	COS
16	*AT4G33070*: Pyruvate decarboxylase, putative	3.43E-12	COS
17	*AT3G17130*: Invertase/pectin methylesterase inhibitor family protein	4.21E-11	COS
18	*AT1G11210*: Similar to unknown protein (TAIR:*AT1G11220.1*)	1.06E-10	COS
19	*AT1G62570*: Flavin-monooxygenase Glucosinolates-Oxygenase 4 (*FMO GS-OX4*)	3.24E-10	COS
20	AT2G16890: UDP-glucoronosyl/UDP-glucosyl transferase family protein	5.52E-10	COS
21	*AT4G25480*: Dehydration response element B1A (*DREB1A*); C-Repeat Binding Factor 3 (*CBF3*)	6.1E-10	COS
22	*AT1G20440*: Cold-Regulated 47 (*COR47*); (*RD17*)	1.08E-09	COS
23	*AT1G61800*: Glucose-6-Phosphate/Phosphate Translocator 2 (*GPT2*)	1.37E-09	Plate
24	*AT4G17550*: Transporter-related	1.52E-09	COS
25	*AT1G62710*: Beta Vacuolar Processing Enzyme (*BETA-VPE*)	5.78E-09	COS

*“ Cold Upregulation Category” indicates whether a gene was upregulated in the cold when plants were grown in soil (“Soil”), on agar plates (“Plate”), on both growth mediums (“COS”), or on neither (“Novel”) in Vogel et al’s study [54].

**Table 3:** The top 25 ranked non-COS genes based on treatment-frequency fold change values at 26 hours

**Rank**	**Annotation**	**Comments**	**P Value (Treatment-Frequency Fold Change, 26 Hours)**	Cold Upregulation Category*[54]
11	*AT1G02820*: Late embryogenesis abundant 3 family protein/LEA3 family protein	LEA family proteins are associated with dehydration stress (and therefore cold) and general environmental stress in plants, and desiccation tolerance in other organisms including bacteria [60]. Cold response genes *COR15ACOR15B* and *COR47* are classified as *LEA* genes. Although not to the same degree as the *COR* genes, expression of this gene was upregulated by cold according to quantitative RT-PCR [60]	3.29E-13	Soil
13	*AT2G23910*: Cinnamoyl-CoA reductase-related	Implicated in the biosynthesis of phenylpropanoids [61,62], which contribute to many different plant responses to biotic and abiotic stress/challenge [63]	9.97E-13	Plate
23	*AT1G61800*: *GPT2* (Glucose-6-Phosphate Translocator 2)	A *gpt2* mutant shows an impairment in photosynthetic acclimation in response to shifts to high irradiance light, which can be exacerbated under cold conditions [64]	1.37E-09	Plate
29	*AT5G06760*: Late embryogenesis abundant group 1 (LEA group 1) domain-containing protein	Similar to other *LEA* above (*AT1G02820*), expression of this gene is upregulated by cold according to quantitative RT-PCR [60]	1.86E-08	Soil
37	*AT3G51240*: *F3H*; *TT6* (Flavanone 3-Hydroxylase; Transparent Testa 6)	Implicated in freezing stress response [65]	7.07E-08	Plate
50	*AT1G60190*: Armadillo/beta-catenin repeat family/U-box domain-containing		1.92E-06	Soil
53	*AT5G24120*: *SIGE/SIG5* (RNA polymerase sigma subunit E); DNA binding/DNA-directed RNA polymerase/sigma/transcription factor	Regulated in blue light by cryptochromes and involved in light-dependent regulation of the photosynthetic apparatus [66]. In a separate study shown to be essential for *Arabidopsis*[67]	3.24E-06	Soil
55	*AT1G10370*: *ATGSTU17/ERD9/*(Early-Responsive to Dehydration 9)	Dehydration responsive [68]	4.43E-06	Plate
57	*AT1G32900*: Starch synthase, putative	Identified in a study on light/cold interactions [69]. Upregulated by cold generally, but upregulated more under cold/light conditions than cold/dark	6.47E-06	**Novel**
60	*AT4G33905*: Peroxisomal membrane protein 22 kDa, putative	Upregulated by stress, including cold treatment [70]	7.71E-06	**Novel**
61	*AT1G01520*: Myb family transcription factor	Upregulated in mutant that has improved freezing tolerance (i.e. *esk1* mutant) [71].	1.63E-05	**Novel**
63	*AT5G57760*: Unknown		1.95E-05	Plate
70	*AT5G14760*: AO (L-aspartate oxidase)	Involved in the synthesis of NAD [72], which is phosphorylated by cold in other plants [73]	4.96E-05	**Novel**
71	*AT1G10585*: Transcription factor	Upregulated under conditions associated with oxidative stress/high light [74]	5.66E-05	Soil
72	*AT5G07010*: Sulfotransferase family	Jasmonate responsive [68]	5.95E-05	Soil
75	*AT2G22590*: Glycosyltransferase family protein	In the same gene family as *UGT91A1*, (a target of a TF that regulates flavonol synthesis), and is thus proposed to impact flavonol biosynthesis, which is a product associated with cold response [75,76]	6.55E-05	Plate
76	*AT3G17609*: *HYH* (HY5-Homolog); DNA binding/transcription factor	Involved in phyB signaling [77]; Required for low temperature-induced anthocyanin accumulation [78]	6.78E-05	**Novel**
81	*AT1G17170*: *ATGSTU24* (*Arabidopsis thaliana* Glutathione S-Transferase (*TAU*) 24)	Member of the Glutathione S-transferase family (involved in flavonoid synthesis and general abiotic stress response) [79]	0.000123	Soil
82	*AT5G07990*: *TT7* (Transparent Testa 7); flavonoid 3′-monooxygenase	Flavonoid biosynthesis protein, which is a product associated with cold response [68,76]	0.000125	Plate
83	*AT3G55940*: Phosphoinositide-specific phospholipase C, putative	Phospholipase C genes, to which this is related, have been associated with responses to stress in *Arabidopsis*[80]	0.000142	Plate
84	*AT3G21560*: *UGT84A2*; UDP-glycosyltransferase/sinapate 1-glucosyltransferase	Upregulated by cold via the phospholipase D-dependent phosphatidic acid production [81]	0.000145	Plate
85	*AT5G49480*: *ATCP1* (CA2 + −Binding Protein 1)	A “cold regulated signaling gene” that is altered in an ice1 mutant background (*ICE1* is a cold/freezing related TF) [51]. Regulation altered under drought conditions [82]. Also (like *UGT84A2*, above) upregulated by cold via phospholipase D-dependent phosphatidic acid production [81]	0.000168	Soil
86	*AT5G44110*: *POP1*	Shown to be upregulated by cold in supplemental table of [83]. Response to Red and Far-Red light via phyA [84]. Also a target of *HY5*[85], which is a transcription factor in light signaling/responsiveness, but also shown to be important for cold dependent anthocyanin accumulation together with *HYH* (above) [78]	0.00017	Soil
87	*AT5G36910*: *THI2.2* (Thionin 2.2); toxin receptor binding	Downregulated under high temperature stress [86], associated with jasmonic acid/salicylic acid signalling [87] and target of *FAR1* and *FHY3*, which function in phyA signaling [88]	0.000174	**Novel**
88	*AT2G31380*: *STH1* (salt tolerance homologue); transcription factor/zinc ion binding, also previously denoted *ZF3*	Like *POP1* above, shown to be upregulated by cold in supplemental table of [83]. Circadian-controlled zinc finger gene with role in light signaling [89]. Additional evidence for role in light signaling and regulation by phytochrome [90,91], and like *THI2* (above), target of *FAR1* and *FHY3*, which function in phyA signaling [88]	0.000176	Soil

*“ Cold Upregulation Category” indicates whether a gene was upregulated in the cold when plants were grown in soil (“Soil”), on agar plates (“Plate”), on both growth mediums (“COS”), or on neither (“Novel”) in Vogel et al’s study [54].

**Figure 6:**
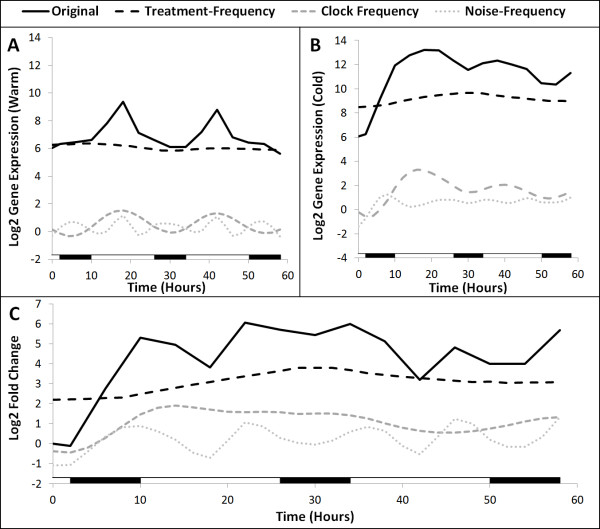
**A case study examining PRIISM output gene expression and fold change data**. The fold change patterns (**A**), warm gene expression patterns (**B**) and cold gene expression patterns (**C**) for the original and PRIISM-processed data for *AtgolS3* (*AT1G09350*), the most highly upregulated gene in response to cold at 26 hours in the treatment-frequency data.

The results of a case study on *ATGolS3* (*AT1G09350*), the gene with the largest fold change in the treatment-frequency data at 26 hours are shown in Figure6. The logged original gene expression curve under warm conditions has a minimum expression level of approximately 6, which is reflected by a flat treatment-frequency expression curve with a nearly constant value of 6 (Figure6A). The rhythmic pattern of the original data in warm conditions is captured in the clock-frequency gene expression curve, and the sharp peaks and sudden changes in slope are captured in the noise-frequency curve (Figure6A). The original gene expression data under cold conditions peaks quite strongly during the first night but retains some cyclical expression. The PRIISM-processed gene expression data shows that the treatment-frequency gene expression is constantly higher in the cold, with a peak at 26 hours, while the clock-frequency gene expression data is only marginally increased, but is increased more in the first day than in the second day (Figure6B, 6C). The fold change graph shown in Figure6C indicates that most of the increase in gene expression is due to treatment-frequency influences for this gene, but the clock-frequency influences upregulate the gene more strongly early in the cold treatment. The noise-frequency fold change pattern matches many of the sharp peaks and valleys in the original fold change pattern, suggesting that much of the noise has indeed been removed (Figure6C).

**Figure 7:**
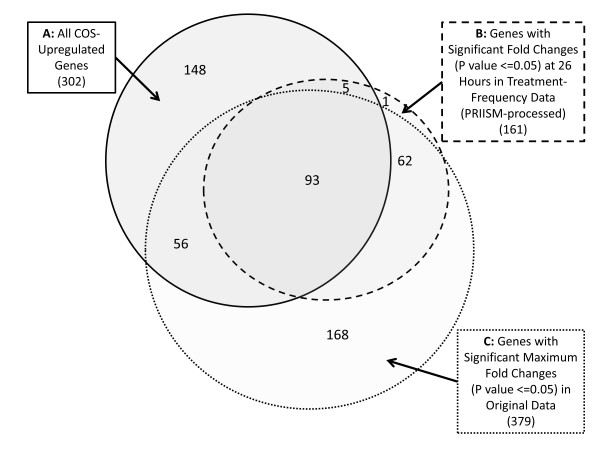
**Venn diagram showing COS-upregulated genes in original and PRIISM-processed significant gene lists**. The number of genes in the overlaps between COS-upregulated genes (**A**) and the significant genes (P value ≤ 0.05) in both the maximum fold change in the original dataset (**B**) and the fold change at 26 hours in the treatment-frequency dataset (**C**) are shown.

To test the statistical significance of PRIISM’s ability to discover treatment-response genes, P-values were calculated using a Z-test for both the maximum fold change from the original dataset and the fold change values at 26 hours in the treatment-frequency dataset. Figure7 shows the number of genes that were found to be significant (P value < =0.05) in these tests, and how many belonged to the COS-upregulated gene list from Vogel et al [54]. Out of the 161 genes significant in the treatment-frequency data at 26 hours, 98 of them (60.9%) were COS upregulated genes, compared to 154 out of 379 (39.3%) for the original dataset.

### Clock-frequency data analysis

**Figure 8:**
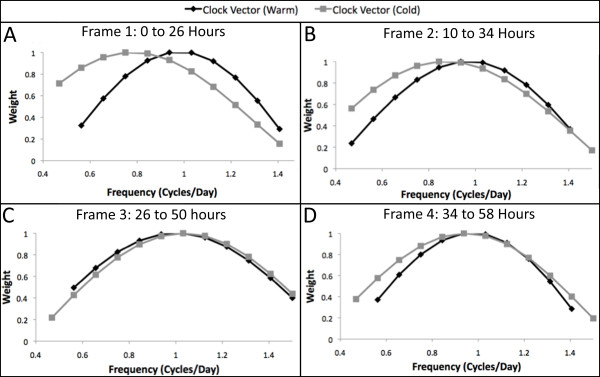
Clock vectors under warm and cold conditions.

The clock vectors calculated by Equation 3 under both warm and cold conditions for each of the time frames are shown in Figure8. The difference between the length and the shape of the warm and cold vectors indicates the circadian rhythm disruption caused by the cold stress. Figure8A shows drastically different frequency profiles for the warm and cold conditions, caused by an abrupt phase shift in the expression data. The clock genes continue to have disrupted frequencies in the second time frame (Figure8B), but appear to return to normal oscillating frequencies, possibly with different phases, in time frames 3 and 4 (Figure8C, 8D).

To study whether the clock-frequency data produced by PRIISM successfully isolated cyclic clock influences from treatment-response influences, the clock-frequency gene expression patterns of eight well-studied cold response genes were matched with standard clock patterns according to the pattern-matching algorithm HAYSTACK [26]. This algorithm (the key component of The Diurnal Project) utilizes a model-based pattern matching algorithm to calculate the phase and cyclic pattern type for each gene in a dataset, and also calculates the correlation of each gene to the closest model, which can be used as an indication of how strong the clock influence is on the gene [26]. HAYSTACK provides *T*-test P-values indicating the probability that an input pattern matches a gene expression model, and provides several types of cyclic clock pattern models to use for comparison [26]. This analysis included the COR genes which have been shown to be under circadian clock control under warm conditions, but gated by cold transcription factors (including the CBF genes) under cold conditions [20]. The results in Table4 indicate that the P values for the clock-frequency gene expression data from PRIISM are substantially lower than the original data (under both warm and cold conditions), often by several orders of magnitude, demonstrating enrichment of clock-frequency gene expression in this data.Note that the remaining portion of the spectrum of the clock-frequency components is simply discarded in PRIISM. In our future work, it will be interesting to further test whether feeding it into the treatment-frequency component will construct more precise results.

**Table 4:** A comparison of the clock patterns between PRIISM-processed and original gene expression data

Gene Name	AGI Number	**P-values for Warm Gene Expression Data**		**P-values for Cold Gene Expression Data**	
		Original	Clock-Frequency (PRIISM)	Original	Clock-Frequency (PRIISM)
*COR15A*	*AT2G42540*	5.6E-17	0	0.125	0.039
*COR15B*	*AT2G42530*	0	0	0.038	4.3E-03
*COR47*	*AT1G20440*	4.7E-09	1.8E-13	0.012	3.8E-03
*COR78*	*AT5G52310*	0	0	0.013	2.3E-03
*CBF1*	*AT4G25490*	5.0E-07	7.2E-08	4.5E-04	3.8E-05
*CBF2*	*AT4G25470*	3.9E-06	1.6E-13	3.2E-08	2.2E-09
*CBF3*	*AT4G25480*	5.5E-14	5.6E-17	2.6E-07	5.0E-10
*RAV1*	*AT1G13260*	1.8E-06	3.0E-10	7.4E-05	2.8E-04
*ZAT12*	*AT5G59820*	3.4E-03	4.9E-05	1.4E-04	1.9E-05

P-values (calculated using the *T*-test HAYSTACK function) indicate the correlation of the gene expression patterns of well-studied cold-responsive genes to pre-defined cyclic clock patterns.

## Conclusions

Circadian rhythm pathways influence the expression patterns of as much as 31% of the *Arabidopsis* genome through complicated interaction pathways, and have been found to be significantly disrupted by biotic and abiotic stress treatments, complicating treatment-response gene discovery methods due to clock pattern mismatches in the fold change statistic. The PRIISM algorithm outlined in this paper is designed to separate pattern changes induced by different forces, including treatment pathways and circadian clock rhythm disruptions. By applying PRIISM on a cold-response dataset, we systematically evaluated our method using maximum fold change and PCA analyses. The results of this study showed that the ranked treatment-frequency fold change analyses produce fewer false positives than the original methodology, and the 26 hour timepoint in the PRIISM produced dataset was the best statistic for distinguishing the most known cold-response genes. In addition, PRIISM also provides gene expression data which represent only circadian clock influences, and may be useful for circadian clock studies. In fact, any existing analysis approach on gene expression data can utilize PRIISM to separate circadian-influenced changes in gene expression. In conclusion, PRIISM is a novel approach for overcoming the problem of circadian disruptions from stress treatments on plants. PRIISM can be integrated with any existing analysis approach on gene expression data to separate circadian-influenced changes in gene expression, and it can be extended to apply to any organism with regular oscillations in gene expression patterns across a large portion of the genome. In future work, we will apply the discrete wavelet transforms (DWT) on higher resolution datasets in order to further enhance the ability of PRIISM to distinguish circadian clock disruption influences from treatment-response pathway influences.

## Competing interests

The authors declare that they have no competing interest.

## Authors’ Contributions

JC, YJ, BR conceived and designed the algorithm. YJ designed MATLAB code for algorithm. BR performed pre-processing and post-processing of data. BR and YJ wrote the manuscript, which was edited by JC, BM, SO and WQ. BM and SO also provided biological knowledge and research of circadian and cold response genes. All authors read and approved the final manuscript.

## Supplementary Material

Additional file 1 **Figure S1: Time frames used to generate FFT results.** Frame sizes and positions are shown in (**A**) and the contribution of each frame to the weighted average at each timepoint is shown in (**B**). **Figure S2: Principal Component Analysis (PCA) Plots.** Principal component analysis (PCA) plots for the original data (**A**) and the first components of the clock-frequency and treatment-frequency data (**B**) are shown. COS-upregulated genes are shown in black circles, COS-downregulated genes (which are not analyzed in detail here) are shown in white diamonds and all other genes are shown as grey dots. **Figure S3:** T**he original, mean-shifted, PRIISM-reconstructed and Butterworth-filter reconstructed gene expression patterns of*AtGolS3.*** (**A**) The original (black) and mean-shifted (grey) expression values of *AtGolS3.* (**B**) Comparison between the treatment-frequency-reconstructed gene expression patterns for *AtGolS3* using PRIISM (Black line) and using a fifth-order Butterworth low-pass filter (grey line). **Figure S4: The frequency spectra of the original, the PRIISM-reconstructed and the Butterworth-filter reconstructed gene expression patterns of*AtGolS3.*** (**A**) The Frequency Spectrum of the original gene expression pattern of *AtGolS3.* (**B**) Comparison of the frequency spectra of *AtGolS3* after processing using PRIISM (white circles) and the fifth-order Butterworth low-pass filter (grey diamonds). The original treatment-frequency spectrum of *AtGolS3* is also shown (red bars). **Figure S5**: **The Bode plot of a fifth-order Butterworth low-pass filter for*AtGolS3***. Click here for file
