# Supplementary material for: Frequency-based time-series gene expression recomposition using PRIISM
Source: BMC Syst Biol. 2012 Jun 15;6:69. doi: 10.1186/1752-0509-6-69 (PMC3464900; doi:10.1186/1752-0509-6-69)
Supplement: Additional file 1 — Figure S1: Time frames used to generate FFT results. Frame sizes and positions are shown in (A) and the contribution of each frame to the weighted average at each timepoint is shown in (B). Figure S2: Principal Component Analysis (PCA) Plots. Principal component analysis (PCA) plots for the original data (A) and the first components of the clock-frequency and treatment-frequency data (B) are shown. COS-upregulated genes are shown in black circles, COS-downregulated genes (which are not analyzed in detail here) are shown in white diamonds and all other genes are shown as grey dots. Figure S3: The original, mean-shifted, PRIISM-reconstructed and Butterworth-filter reconstructed gene expression patterns ofAtGolS3. (A) The original (black) and mean-shifted (grey) expression values of AtGolS3. (B) Comparison between the treatment-frequency-reconstructed gene expression patterns for AtGolS3 using PRIISM (Black line) and using a fifth-order Butterworth low-pass filter (grey line). Figure S4: The frequency spectra of the original, the PRIISM-reconstructed and the Butterworth-filter reconstructed gene expression patterns ofAtGolS3. (A) The Frequency Spectrum of the original gene expression pattern of AtGolS3. (B) Comparison of the frequency spectra of AtGolS3 after processing using PRIISM (white circles) and the fifth-order Butterworth low-pass filter (grey diamonds). The original treatment-frequency spectrum of AtGolS3 is also shown (red bars). Figure S5: The Bode plot of a fifth-order Butterworth low-pass filter forAtGolS3. [file 1752-0509-6-69-S1.doc]

# Supplemental Information for “Frequency-Based Time-Series Gene Expression Recomposition using PRIISM”

A. Supplemental Figures Referenced in the Manuscript


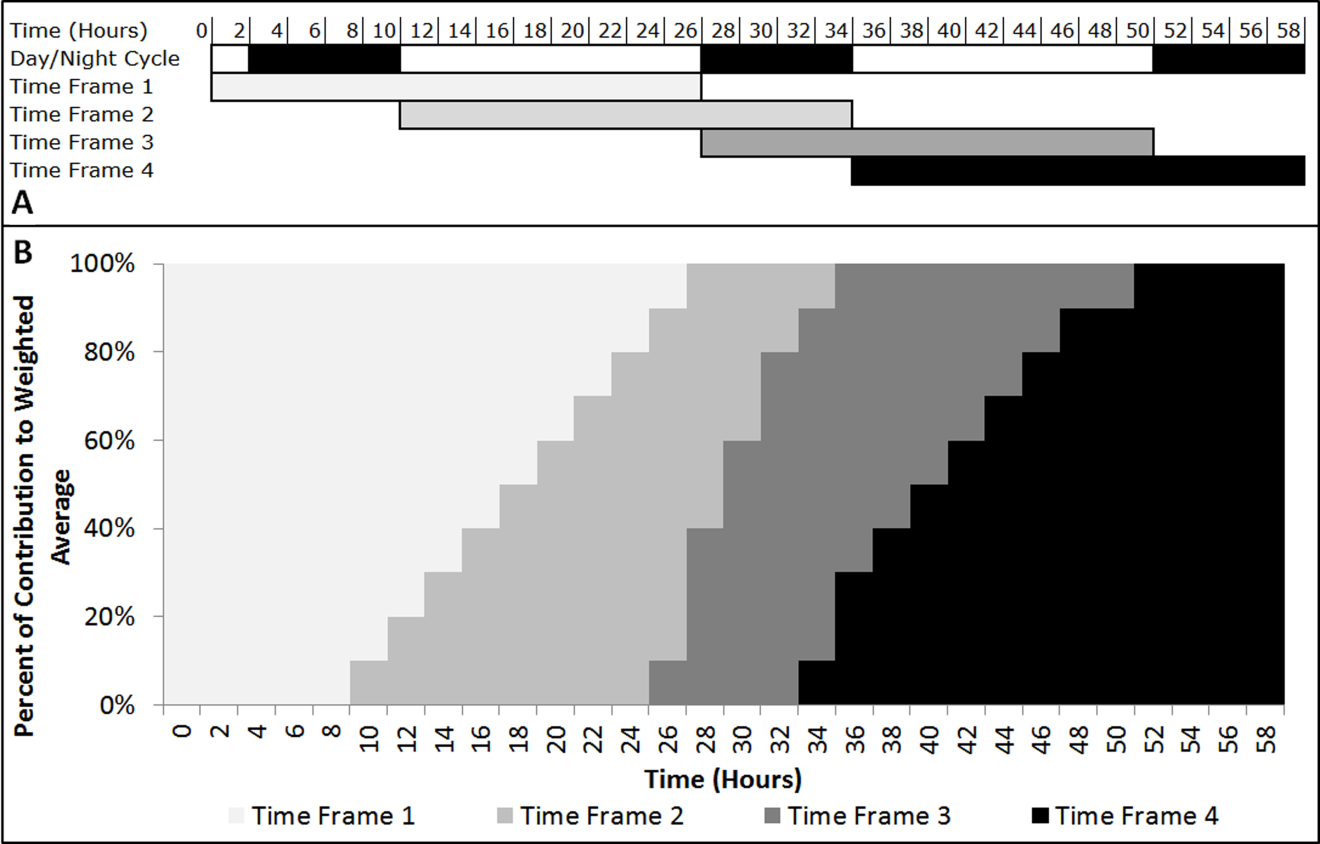


**Figure S1**: Time frames used to generate FFT results. Frame sizes and positions are shown in (A) and the contribution of each frame to the weighted average at each timepoint is shown in (B).


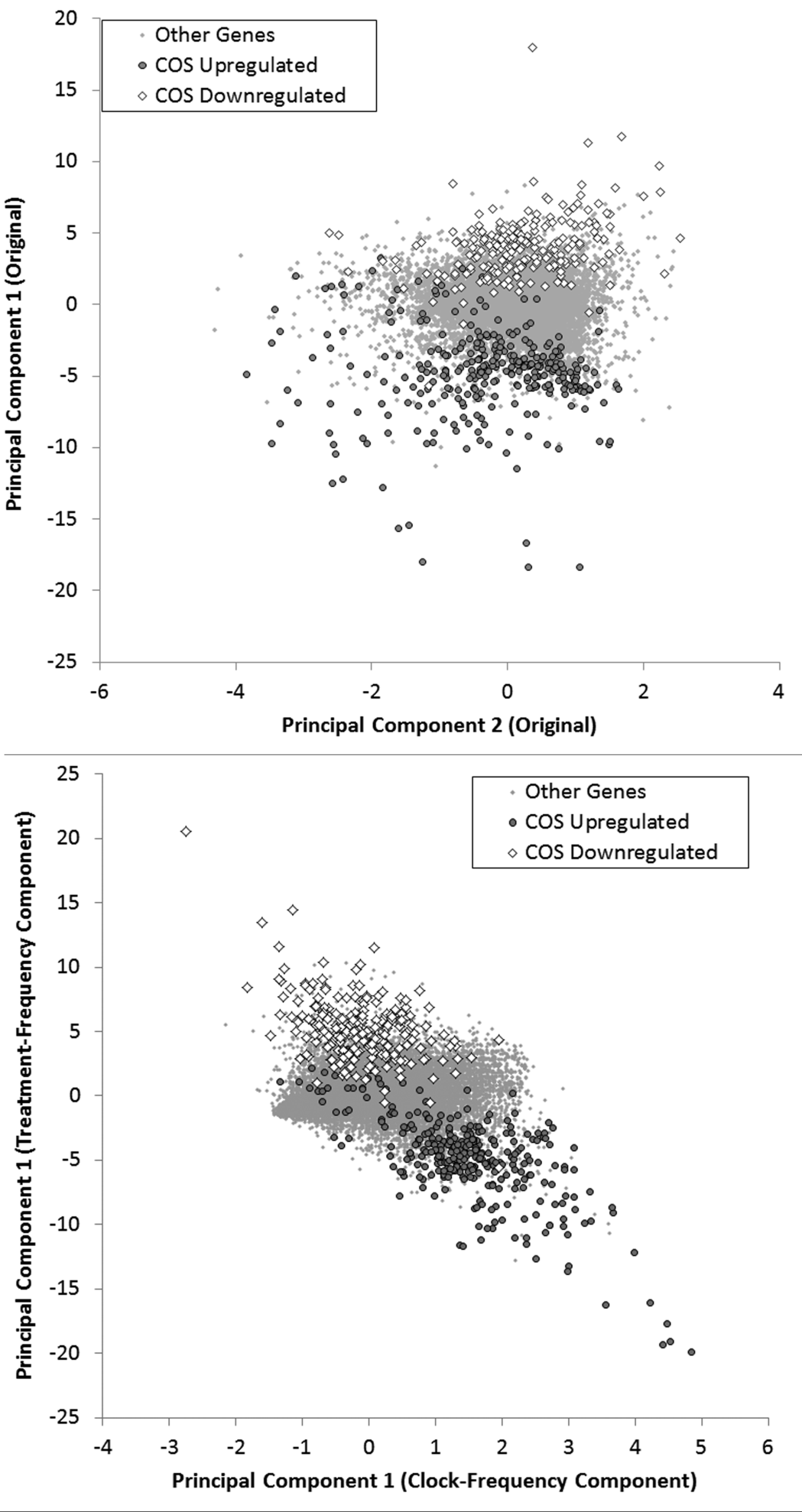


**Figure S2**: Principal component analysis (PCA) plots for the original data (A) and the first components of the clock-frequency and treatment-frequency data (B). COS-upregulated genes are shown in black circles, COS-downregulated genes (which are not analyzed in detail here) are shown in white diamonds and all other genes are shown as grey dots.

B. Justification for choosing a steep cut-off frequency for the low-pass filter

When designing a low-pass filter, the balance between approximating desired frequency response and reducing ringing artifacts should be considered. To better explain why we used an ideal low-pass filter with a steep cutoff rather than a Butterworth filter for treating treatment-frequency components, we will use the gene *AtGolS3* (*AT1G09350*) as a case study. The original gene expression values and the mean-shifted values of *AtGolS3* are shown in Fig. S3A.


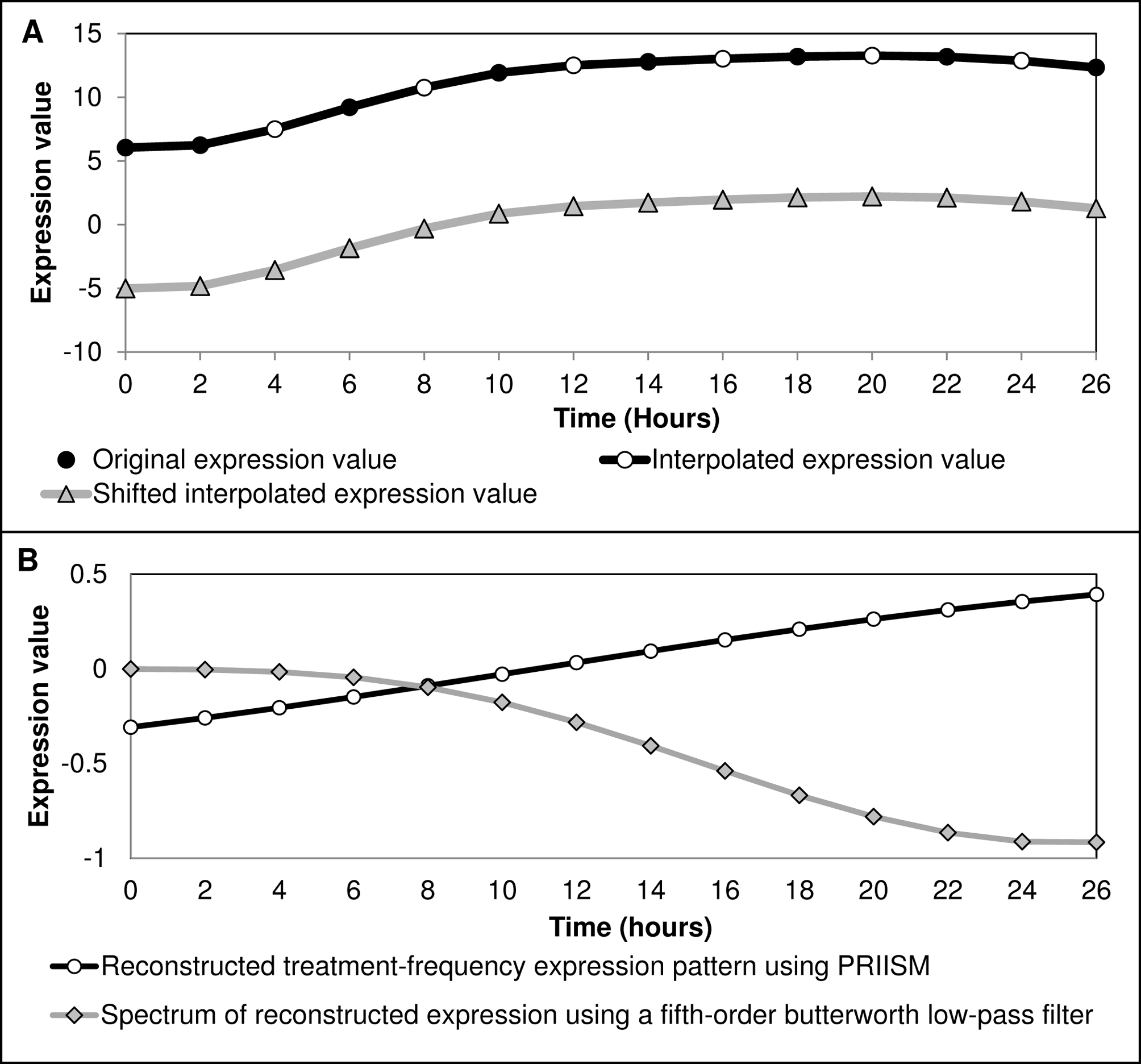


**Figure S3**: The original, mean-shifted, PRIISM-reconstructed and Butterworth-filter reconstructed gene expression patterns of *AtGolS3.* (A) The original (black) and mean-shifted (grey) expression values of *AtGolS3.* (B) Comparison between the treatment-frequency-reconstructed gene expression patterns for *AtGolS3* using PRIISM (Black line) and using a fifth-order Butterworth low-pass filter (grey line).

In PRIISM, the magnitude spectrum of the gene expression is derived by applying Fast Fourier Transform (FFT) on the shifted interpolated values (Fig. S4A). In this figure, the spectrum of the low-frequency components is highlighted using red bars.


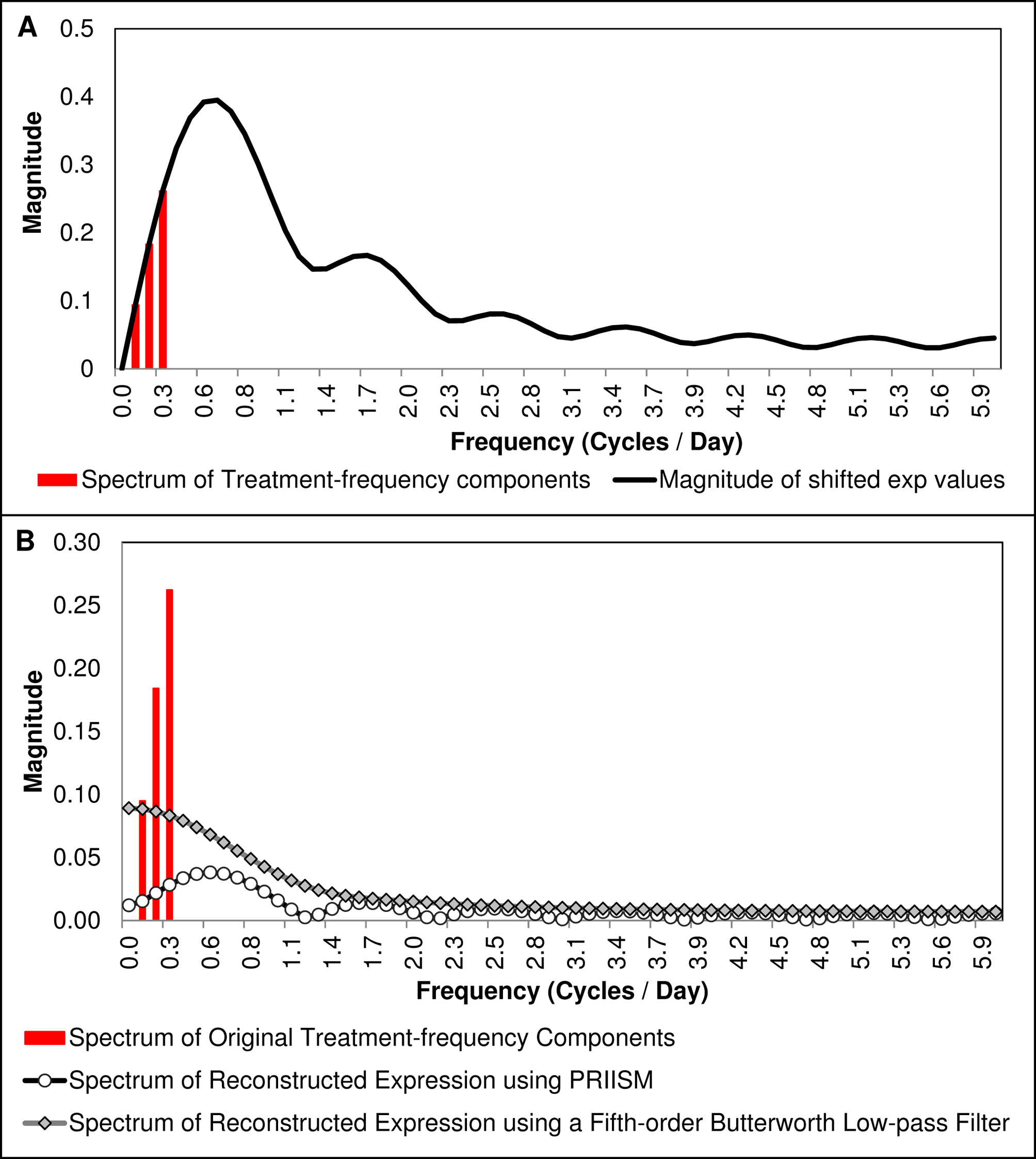


**Figure S4**: The frequency spectra of the original, the PRIISM-reconstructed and the Butterworth-filter reconstructed gene expression patterns of *AtGolS3.* (A) The Frequency Spectrum of the original gene expression pattern of *AtGolS3.* (B) Comparison of the frequency spectra of *AtGolS3* after processing using PRIISM (white circles) and the fifth-order Butterworth low-pass filter (grey diamonds). The original treatment-frequency spectrum of *AtGolS3* is also shown (red bars).

In PRIISM, an ideal low-pass filter with a steep cut-off frequency was used to reconstruct the low-frequency expression pattern. The resulting expression pattern is the black line in Fig. S3B. Alternatively, if we replace the ideal low-pass filter with a fifth-order Butterworth filter (Fig. S5), the result is the reconstructed treatment-frequency expression pattern shown in the grey line (grey diamonds) in Fig. S3B.


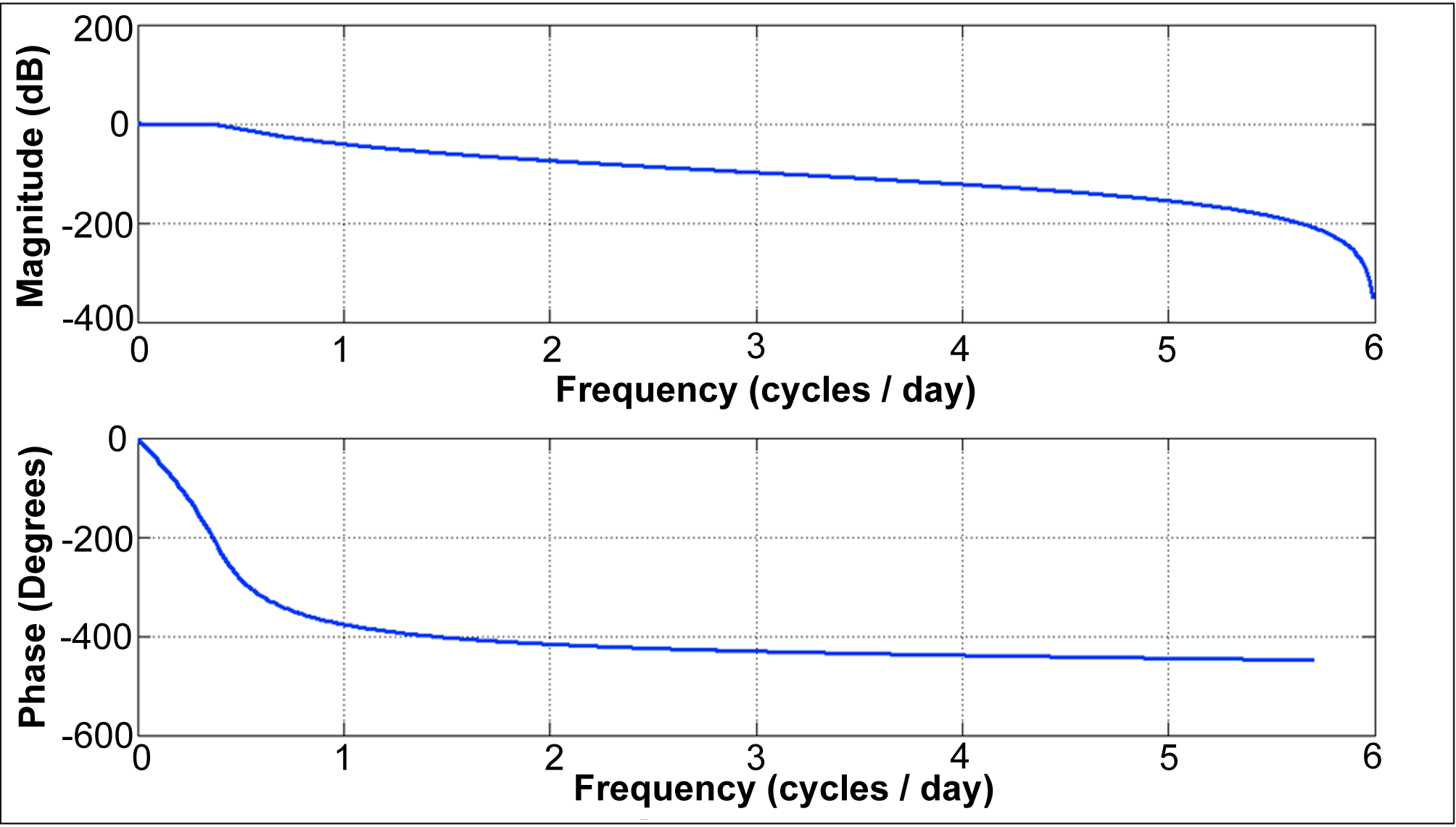


**Figure S5**: The Bode plot of a fifth-order Butterworth low-pass filter for *AtGolS3.*

In order to further evaluate the reliability of the reconstructed expression patterns, we have computed the spectra of the two reconstructed series by performing FFT again. The two curves in Fig. S4B are the spectra of the reconstructed patterns by PRIISM and a Butterworth low-pass filter respectively. The red bars in Fig. S4B represent the magnitudes of the treatment-frequency components in the spectrum of the original expression values, which is the desired spectrum of the reconstructed treatment-frequency expression pattern.

Ringing artifacts (the phenomenon of output oscillating near a sharp transition in the input) appear as a result of the ideal low-pass filter that is used in PRIISM. Therefore, the spectrum of the reconstructed pattern by PRIISM peaks at high frequencies. But the heights of the peaks are relatively small because gene expression values usually do not change sharply. Consequently, there is no visible oscillation in the reconstructed pattern in Fig. S3B.

Note that the energy in low frequency band is very small because the low-pass bandwidth is small and the expression values were adjusted to zero mean before applying FFT. The purpose of the signal decomposition is mainly for capturing the changing trend of the treatment-frequency expression pattern.

Because of the Parseval's theorem [91], the magnitude of the spectrum of the reconstructed gene expression values using PRIISM is much smaller than that of the desired spectrum. However, their magnitudes at the corresponding frequencies are proportional to each other. Therefore, the reconstructed gene expression patterns using PRIISM (Fig. S3B) has a similar changing trend to the original one.

Because the low pass bandwidth is relatively narrow, the transition band in a Butterworth filter could worsen the frequency response. As a result (Fig. S4B), the spectrum obtained by a Butterworth filter is totally different from the desired spectrum. Consequently, the reconstructed gene expression pattern (Fig. S3B) goes down, which is opposite to the original pattern (Fig. S3A).

In conclusion, in the special case of PRIISM (for which only low-resolution datasets are available), more artifacts were added by using the Butterworth filter compared with the simple ideal low-pass filter. Therefore, we simply applied an ideal low-pass filter with a steep cutoff frequency rather than a Butterworth filter for treating treatment-frequency components. However, a tapering filter was used for the Circadian Clock Frequency Range (CCFR), which has more frequency values to use as input. We will continue to work on finding and applying the best possible filters in future versions of PRIISM.
